# New Appliances and Things Medical

**Published:** 1897-03-20

**Authors:** 


### NEW APPLIANCES AND THINGS MEDICAL.

**[We shall be glad to receive, at our Office, 28 & 29, Southampton Street, Strand, London, W.C., from the manufacturers, specimens of all new preparations and appliances which may be brought out from time to time.]**

### LACTOMALTINE.

(The Lactomaltine Company, 35, Snow Hill, E.C.)

This excellent preparation has b.en submitted to our notice, and after careful trial we have found it in each case readily taken by the patient, and digested and absorbed in cases of acute dyspepsia. It contains the elements of malt extract, and milk rich in cream. It is within limits a perfect food, that is to say, it contains representatives of the three necessary food substances—proteids, carbohydrates, and fats. Its main feature, however, is that these principles are combined in an agreeable manner, and constitute a mixture which is as readily digested and absorbed, and it is taken by patients of most capricious tastes. Its use is specially indicated in those cases of malnutrition in which cod liver oil is usually prescribed. As a food of fattening qualities we regard it as a most desirable substitute for the latler oil.

### FROMM'S VEGETABLE EXTRACT.

(Fromm's Extract Company, 61½, Fore Street, E.C.)

In the preparation of this new extract the above company have made a completely new departure in relying on the vegetable kingdom for the material from which to manufacture a most palatable extract. Analysis shows that it contains representatives of the proteid, carbohydrate, and fatty groups, and a large proportion of those salts which are most important for the maintenance of health. Further than this, physiological test demonstrates that it is very readily and completely digested. Briefly, then, Fromm's Extract may be regarded as a very valuable and condensed food. It may be used in conjunction with soups, gravy, jellies, sauces, hashes, &c., adding to their nutritive value and flavour. To those of gouty tendency we strongly recommend Fromm's Extract, since proteid matter of vegetable origin appears to suit such constitutions far better than when it is of an animal nature.

### MELLIN'S INFANT'S FOOD AND EMULSION OF COD LIVER OIL AND HYPOPHOSPHITES.

(Mellin's Food Co., Peckham, London.)

The first-named preparation is now so universally known, and so generally recognised as one of the most valuable adjuncts to infant feeding that no further mention is here necessary. The emulsion of cod liver oil and hypophosphites, though equally valuable, has a less extensive use. It is an emulsion of the very finest variety, consisting of about 50 per cent. of oil and containing a rich percentage of hypophosphites. From personal experience we can testify that it is far more readily taken than the oil in the simple form, and being already emulsified the digestive processes are more than half completed before entering the stomach, hence it is readily absorbed, and the chance of unpleasant eructation after its exhibition reduced to a minimum. We regard it as the finest form in which cod liver oil can be administered.

### "PURO" TEA.

(The Shanghai and Foochow Tea Association).

In our last issue, in reporting our favourable opinion of "Puro" tea, we inadvertently omitted to state that the proprietors were the Shanghai and Foochow Tea Association, 22, Fenchurch Street, E.C.

